# The roles of exercise stress echocardiography for the evaluation of heart failure with preserved ejection fraction in the heart failure pandemic era

**DOI:** 10.1007/s10396-024-01468-2

**Published:** 2024-06-26

**Authors:** Naoki Yuasa, Tomonari Harada, Kazuki Kagami, Hideki Ishii, Masaru Obokata

**Affiliations:** Department of Cardiovascular Medicine, Gunma University Graduate School of Medicine, 3-39-22 Showa-Machi, Maebashi, Gunma 371-8511 Japan

**Keywords:** Aging, Dyspnea clinics, Exercise testing, Heart failure, Stress echocardiography

## Abstract

Heart failure with preserved ejection fraction (HFpEF) accounts for nearly 70% of all HF and has become the dominant form of HF. The increased prevalence of HFpEF has contributed to a rise in the number of HF patients, known as the “heart failure pandemic”. In addition to the fact that HF is a progressive disease and a delayed diagnosis may worsen clinical outcomes, the emergence of disease-modifying treatments such as sodium-glucose transporter 2 inhibitors and glucagon-like peptide-1 receptor agonists has made appropriate and timely identification of HFpEF even more important. However, diagnosis of HFpEF remains challenging in patients with a lower degree of congestion. In addition to normal EF, this is related to the fact that left ventricular (LV) filling pressures are often normal at rest but become abnormal during exercise. Exercise stress echocardiography can identify such exercise-induced elevations in LV filling pressures and facilitate the diagnosis of HFpEF. Exercise stress echocardiography may also be useful for risk stratification and assessment of exercise tolerance as well as cardiovascular responses to exercise. Recent attention has focused on dedicated dyspnea clinics to identify early HFpEF among patients with unexplained dyspnea and to investigate the causes of dyspnea. This review discusses the role of exercise stress echocardiography in the diagnosis and evaluation of HFpEF.

## Introduction

Heart failure (HF) represents a significant public health problem, with a worldwide prevalence of over 64 million [1]. It is estimated that there are 1.2 million patients with heart failure in Japan (prevalence rate of approximately 1%), and the number is projected to increase to 1.3 million by 2035 despite a reduction in overall population, indicating a pandemic of HF [2]. The prevalence of heart failure with preserved ejection fraction (HFpEF) compared with HF with reduced ejection fraction (HFrEF) is increasing, possibly due to the aging of the general population and the increasing burden of cardiac and metabolic comorbidities. Serial results from the chronic heart failure analysis and registry in the Tohoku District (CHART) showed a clear trend of increasing prevalence of HFpEF from 50.6% in CHART-1 (2000–2005) to 68.7% in CHART-2 (2006–2010) [3]. Many patients with HF, especially those with HFpEF, are diagnosed at the first hospitalization event for decompensated HF. This may lead to delayed therapeutic intervention and poor clinical outcomes, with approximately one in four rehospitalizations and one in five all-cause deaths within 1 year in Japan [4]. In addition, deterioration of HF can impair activities of daily living and reduce cognitive function, which substantially impairs the quality of life of afflicted patients [5].

As HF is progressive and irreversible, there has been a paradigm shift towards early identification [6, 7]. The American Diabetes Association recommends the measurement of natriuretic peptides (NPs) in patients with type 2 diabetes (T2DM), with use of relatively lower cutoff values of B-type natriuretic peptide (BNP) > 50 pg/mL or N-terminal pro-B-type natriuretic peptide (NT-proBNP) > 125 pg/mL [8]. Similarly, the Japanese Heart Failure Society recommends evaluation or referral to a cardiologist for patients with BNP > 35 pg/mlL or NT-proBNP > 125 pg/mL [9]. Compared to HFrEF, the diagnosis of HFpEF is challenging, particularly in cases without overt congestion [6, 10]. Exercise stress echocardiography may be a useful diagnostic tool in identifying HFpEF in such patients, garnering increased interest in recent years [11–14]. In this review article, we discuss the roles of exercise stress echocardiography in the diagnostic evaluation of HFpEF, highlighting the importance of early identification.

### Diagnostic approach to HFpEF

Although the diagnosis of HFpEF among patients with apparent pulmonary or systemic congestion is straightforward, its identification among those with euvolemia is challenging [6, 10]. Exertional dyspnea or fatigue represents a common manifestation in such patients, but these symptoms are not specific to patients with HF. Similar symptoms may be seen in patients with non-cardiac conditions, such as lung disease, anemia, severe obesity, renal disease, liver disease, or deconditioning. Therefore, the first step in diagnosing HFpEF among patients with exertional dyspnea is to exclude non-cardiac mimics [15]. To achieve this, detailed medical history, blood tests (NPs, blood cell counts, liver and kidney function, and D-dimer), chest X-rays, electrocardiograms, and standard echocardiography should be performed. Measurements of NPs are useful for excluding the presence of HF because of their high sensitivity [16]. However, compared to patients with HFrEF, NP levels are often lower in patients with HFpEF, particularly in those with obesity [17–20]. Of note, it is reported that clinical outcomes are poor even in patients with HFpEF and relatively low NT-proBNP levels [21, 22].

Echocardiography plays an essential role in the diagnosis of HFpEF [10, 23, 24]. The diagnosis of HFrEF is straightforward because it can be identified by demonstrating a reduced LVEF among patients with symptoms of HF. However, there are diagnostic challenges in cases of HFpEF where LVEF is preserved, making it difficult to distinguish whether the cause of dyspnea is HFpEF or a non-cardiac condition. As per the universal definition of HF [25], the presence of fluid retention or congestion on chest X-ray makes the diagnosis of HFpEF easy, but in the early stages, overt signs of congestion may be lacking. In such cases, echocardiographic parameters of LV diastolic dysfunction are used to identify objective evidence of cardiac congestion, or elevated left atrial (LA) pressure, and these include transmitral flow (TMF) pattern, early diastolic mitral tissue (e’) velocity, the ratio of early diastolic mitral inflow velocity to e’ velocity (E/e’ ratio), tricuspid regurgitation velocity (TRV), and pulmonary venous flow pattern [14, 26]. While these indices have high specificity to identify elevated LA pressure, their sensitivity is generally poor. It has been reported that E/e’ ratio is poorly sensitive (sensitivity 0–70%) to detect elevated LV filling pressures in patients with normal EF [23]. The American Society of Echocardiography and the European Association of Cardiovascular Imaging (ASE/EACVI) recommends a combination of multiple echocardiographic parameters for evaluating LA pressure to complement the limitations of low sensitivities of individual LV diastolic dysfunction indices, but even this has been reported to have low sensitivity [6, 26–28]. These data suggest that many patients with HFpEF will be missed if relying solely on echocardiographic parameters.

The primary reason for this may be related to the fact that LV filling pressure is often normal at rest in patients with HFpEF and no or modest congestion [29, 30]. Right heart catheterization revealed that 44% of patients with HFpEF presenting with chronic dyspnea and less congestion had normal pulmonary capillary wedge pressure (PCWP) at rest [6]. Of note, many patients with HFpEF develop abnormal increases in LV filling pressure during physiological stress such as exercise, which highlights the need for exercise stress testing for the evaluation of HFpEF. Exercise stress echocardiography estimates the increase in LV filling pressure and is often used as the initial test due to its noninvasive nature [6, 14]. When should stress echocardiography be considered?

### Indications for exercise stress echocardiography

In the diagnostic work-up, the probability of HFpEF (pre-test probability) can be assessed based on resting assessments [6]. Patients with an intermediate pre-test probability are likely to be candidates for exercise stress echocardiography [6, 14]. In contrast, exercise echocardiography is not required for patients with a low (e.g., a young patient with no metabolic comorbidities and low NP levels) or high pre-test probability (e.g., a patient with high NP levels, pulmonary congestion on chest x-ray, and enlarged LA on echocardiography). The H_2_FPEF score and the HFA-PEFF score can be used to determine the pretest probability [14, 31, 32]. Although the H_2_FPEF score is an evidence-based scoring system developed using a gold standard test of invasive hemodynamics exercise testing (invasive cardiopulmonary exercise testing), its applicability to Japanese patients remains controversial due to differences in clinical characteristics from Westerners [33]. For example, the H_2_FPEF score weights 2 points for BMI of ≥ 30 kg/m^2^, whereas the prevalence of BMI ≥ 30 kg/m^2^ is reported to be rare in Japanese patients with HFpEF (~ 6.5%) [34, 35]. Echocardiography is often not available to primary care physicians, and the H_2_FPEF score and the HFA-PEFF algorithm, which include echocardiographic indices, may not be used. Thus, a scoring system using simplified indices is needed.

### Case presentation

*A 67-year-old woman was referred for exercise stress echocardiography for the evaluation of dyspnea. She was obese (BMI 27.5 kg/m*^*2*^) *and had systemic hypertension, dyslipidemia, and a history of persistent atrial fibrillation (AF) 2 years previously, which was treated with catheter ablation. There was no evidence of systemic congestion, such as peripheral edema or jugular vein distention. Her chest X-ray showed cardiomegaly (cardiothoracic ratio 55%), and NT-proBNP levels were modestly elevated at 184 pg/mL. Transthoracic echocardiography showed a normal LVEF (60–65%), LA volume index of 32 ml/m*^*2*^, *and normal TRV of 2.5 m/sec. Transmitral flow demonstrated a normal or peudonormal pattern, with a borderline E/e' ratio of 12.7.*

*Despite the assessment of normal LA pressure based on resting echocardiographic findings, a diagnosis of HFpEF could not be excluded because of typical HF symptoms such as shortness of breath on exertion, a history of AF and hypertension, cardiac enlargement on chest X-ray, and mildly elevated NP levels. Thus, exercise stress echocardiography was warranted for further evaluation of her symptoms of dyspnea.*

*Exercise stress echocardiography was performed because of the intermediate probability of having HFpEF (H*_*2*_*FPEF score: 5 points, HFA-PEFF score: 3 points)*.

### Exercise stress echocardiography in clinical practice

Rather than pharmacological stress, exercise stress should be performed in any patient capable of physical exercise as this provides detailed information regarding the response of the cardiovascular system to various changes induced by exercise, such as increases in preload and biventricular systolic and diastolic function, chronotropic response, and afterload reduction [36]. Exercise stress echocardiography can be performed using either a bicycle ergometer, treadmill, or other type of protocol such as handgrip exercise [37]. The guidelines from the ASE/EACVI recommend a supine ergometer exercise protocol for the evaluation of HFpEF (i.e., diastolic stress echocardiography) [36]. The greatest advantage of ergometer exercise compared to treadmill exercise is that it allows the continuous acquisition of echocardiographic images throughout the test. By adjusting the workload, it can be performed in elderly patients, which is common in HFpEF, with a very low risk of falling [11, 38]. On the other hand, exercise is usually performed in the standing position in daily living, and the semi-supine ergometer may be less physiological in terms of posture [39, 40]. Some abnormalities such as preload insufficiency or LV outflow tract obstruction may be masked in the supine position [41]. The ASE/EACVI guidelines recommend a stepwise protocol starting at a workload of 25 watts (W) and increasing the intensity by 25 W every 3 min [36]. Blood pressure, heart rate, oxygen saturation, electrocardiographic changes, and symptoms should be monitored throughout the exercise. The incidence of complications has been reported to be less than 0.2%, including congestive heart failure and arrhythmias [42].

The ASE/EACVI guidelines advocate the acquisition of TMF, mitral e' velocity, and TRV during exercise for the assessment of unexplained dyspnea and HFpEF [26, 36]. The E/e’ ratio is a key parameter that estimates LV filling pressure during exercise [6, 43]. Exercise E/e' ratio > 15 may suggest an elevated LV filling pressure [6]. However, if TMF velocities are fused during elevated heart rate, it is no longer measurable. In such cases, a normal E/e’ ratio during low-level exercise (20 W) may be used to rule out HFpEF [30]. Alternatively, E/e’ ratio obtained at a submaximal workload (heart rate: 100–110 beats/min) or after exercise can be used [44]. Tricuspid regurgitation velocity is another important parameter during exercise stress echocardiography as it may reflect exercise PH secondary to elevation in LV filling pressures [45]. Tricuspid regurgitant velocity represents the right atrial (RA)-right ventricular pressure gradient and does not account for RA pressure [45]. Note that pulmonary artery pressure may be underestimated in HFpEF with markedly elevated RA pressure, such as in obesity or severe TR [17]. In this light, peripheral venous pressure may provide an accurate estimation of RA pressure during exercise [46]. Note that elevated TRV alone is not sufficient to differentiate HFpEF from pulmonary hypertension, especially in patients at risk for pulmonary hypertension, such as those with connective tissue disease, venous thromboembolism, or pulmonary diseases [36]. Another limitation may be the low feasibility of obtaining high-quality TR envelopes during exercise [6, 47].

**Fig. 1:**
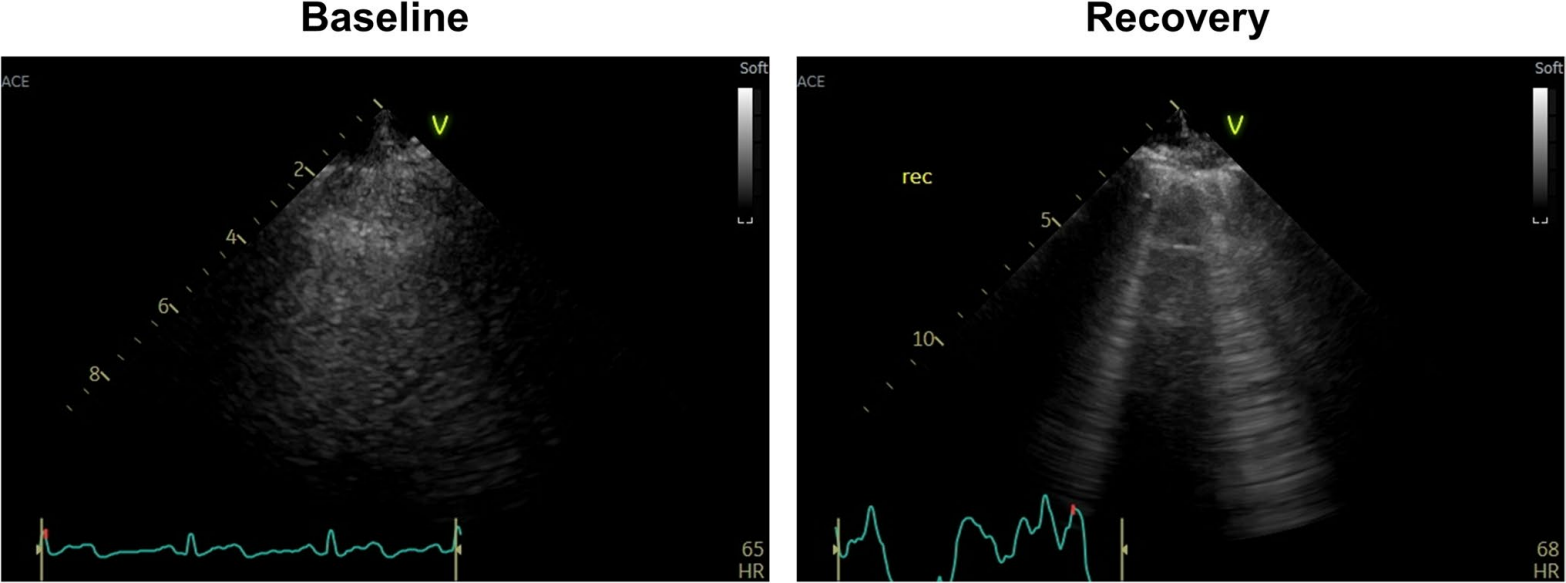
Lung ultrasound to identify ultrasound B-lines during exercise stress echocardiography. Ultrasound B-lines are absent at baseline but develop in the recovery phase in a patient with heart failure with preserved ejection fraction (HFpEF). Ultrasound B-lines are laser-like hyperechoic lines that originate from the pleural line and extend to the bottom of the ultrasound screen

To complement these conventional parameters, new indices have emerged to identify elevated LV filling pressures during exercise. Lung ultrasound can visualize lung congestion as ultrasound B-lines, which represent vertical, hyperechoic lines that originate from the pleural line in patients with HF. Combining lung ultrasound and exercise echocardiography allows the identification of exercise-induced lung congestion in patients with HFpEF (Fig. 1) [48, 49]. It has been reported that ultrasound B-lines are increased throughout exercise and are most prominent during the recovery period in patients with HFpEF [50]. This may increase the feasibility of obtaining B-lines. LA dysfunction is common in patients with HFpEF, possibly through chronic elevation in LV filling pressure and AF burden [51]. Multiple studies have demonstrated that LA reservoir strain at rest quantifies the severity of LA dysfunction and provides the diagnostic ability for HFpEF over E/e’ ratio [51–54]. A recent study has shown a superior diagnostic value of combined assessment of exercise LA reservoir strain and E/e’ ratio to exercise E/e’ alone for the diagnosis of HFpEF [55].

**Fig. 2:**
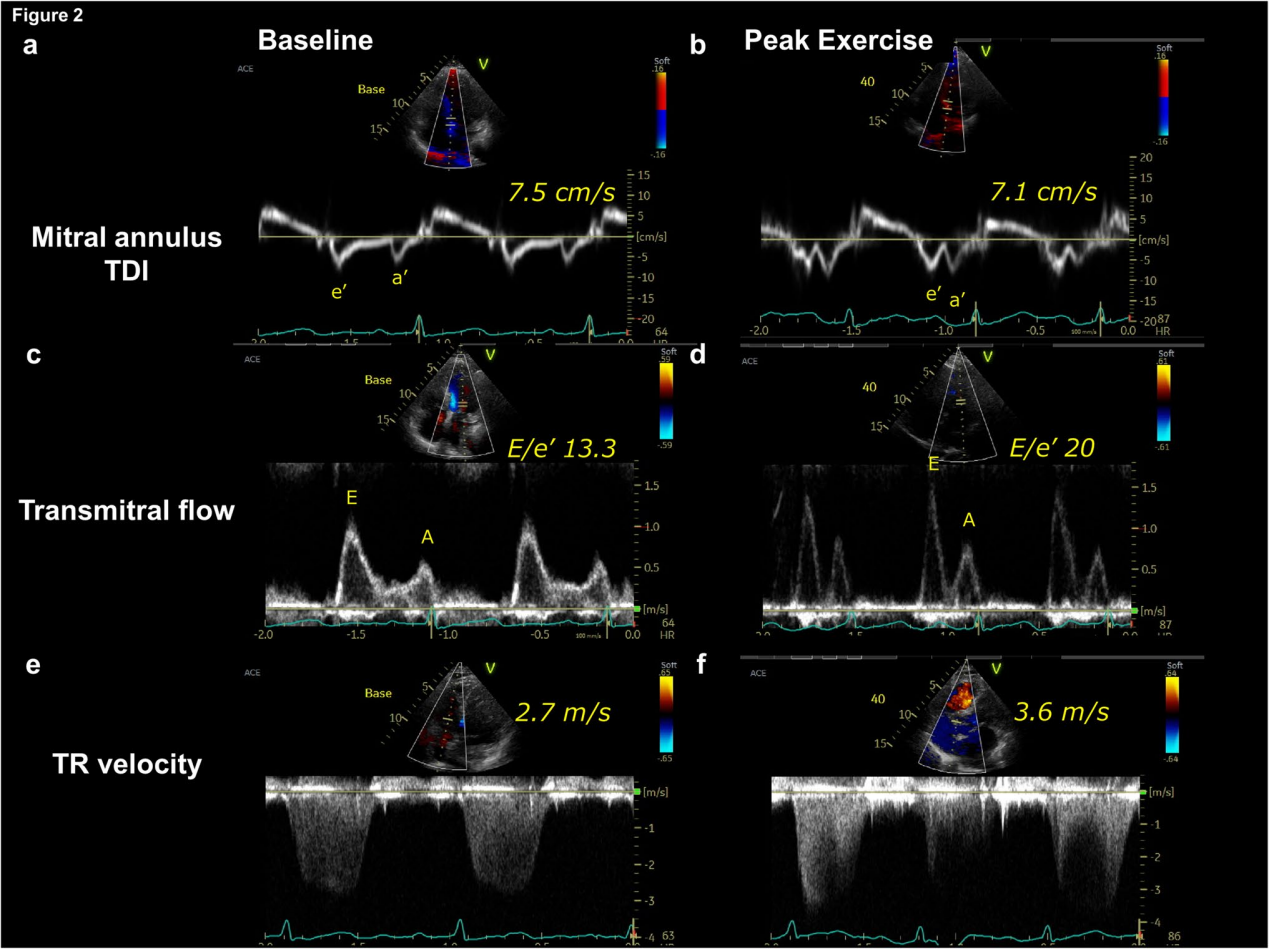
A representative case. (**a**, **b**) Early diastolic mitral annular tissue velocity (e’) did not increase during exercise. (**c**, **d**) Transmitral flow pattern demonstrated a marked increase in early diastolic mitral inflow velocity (E-wave) during exercise, resulting in elevation of the E/e’ ratio from 13.3 to 20.0. (**e**–**f**) Tricuspid regurgitation velocity increased from 2.7 m/sec to 3.6 m/sec during exercise

*Mitral tissue Doppler imaging showed an absence of increase in early diastolic mitral annular tissue velocity (e’) during exercise (Fig.* 2*a, b). The TMF pattern revealed a marked increase in mitral E-wave during exercise, resulting in elevation of the E/e’ ratio from 13.3 to 20.0 (Fig. *2*c, d). Tricuspid regurgitation velocity also increased from 2.7 m/sec to 3.6 m/sec (Fig. *2*e, f). Lung ultrasound revealed that ultrasound B-lines increased from two lines at rest to four lines during post-exercise. Simultaneous expired gas analysis showed a marked reduction in peak oxygen consumption of 9.1 mL/min/kg.*

### Diagnosis of HFpEF using exercise stress echocardiography

Currently, two diagnostic criteria are available: the ASE/EACVI and the HFA-PEFF algorithm [26]. The ASE/EACVI criteria require all three of the following to diagnose the presence of elevated LV filling pressure: elevations in E/e' ratio (average E/e’ > 14 or septal E/e’ ratio > 15) and TRV during exercise (peak TRV > 2.8 m/sec) and low e' at baseline (septal e’ < 7 cm/sec or lateral e’ < 10 cm/sec) [26]. This strict requirement may result in low sensitivity to diagnosis HFpEF [6]. The HFA-PEFF algorithm recommends a multi-step approach in which exercise stress echocardiographic findings (average E/e' ratio > 15, TR velocity > 3.4 m/sec) are added to the score calculated from the resting echocardiography and NPs to diagnose HFpEF [14]. It should be noted that both schemes are based on expert opinions. Thus, evidence-based criteria are warranted to accurately diagnose HFpEF based on exercise stress echocardiography. Ideally, such criteria should be developed by definitive ascertainment of HFpEF or non-cardiac dyspnea using the gold standard of invasive hemodynamic exercise testing.

Invasive hemodynamic exercise testing may be required to diagnose or rule out HFpEF in some cases with an equivocal or non-diagnostic exercise echocardiographic result or concern for pulmonary arterial hypertension [6, 14, 31, 32, 56, 57]. The greatest advantage of invasive hemodynamic exercise testing is the ability to directly measure intracardiac pressures at rest and during exercise, but there are increased costs, the requirement for specialized equipment and operator expertise, and measurable risk [6].

*This case met the criteria for a diagnosis of HFpEF based on the HFA-PEFF algorithm. A sodium-glucose cotransporter 2 (SGLT2) inhibitor was initiated.*

### Roles of exercise stress echocardiography beyond the diagnosis

In addition to diagnosis of HFpEF, exercise stress echocardiography may provide several potentially important clinical implications. The identification of HFpEF with exercise stress echocardiography may allow risk stratification in patients with chronic exertional dyspnea. Our group showed that patients diagnosed with HFpEF based on the HFA-PEFF algorithm had a seven-fold increased risk of composite events of all-cause mortality or worsening HF events than those who did not meet the HFpEF criteria [7]. Of note, patients with HFpEF who received guideline-directed medical treatment after the diagnosis had a lower composite endpoint than those who did not [7]. Although this was a retrospective observational study, these data suggest a potential benefit of early diagnosis and therapeutic intervention in HFpEF.

**Fig. 3:**
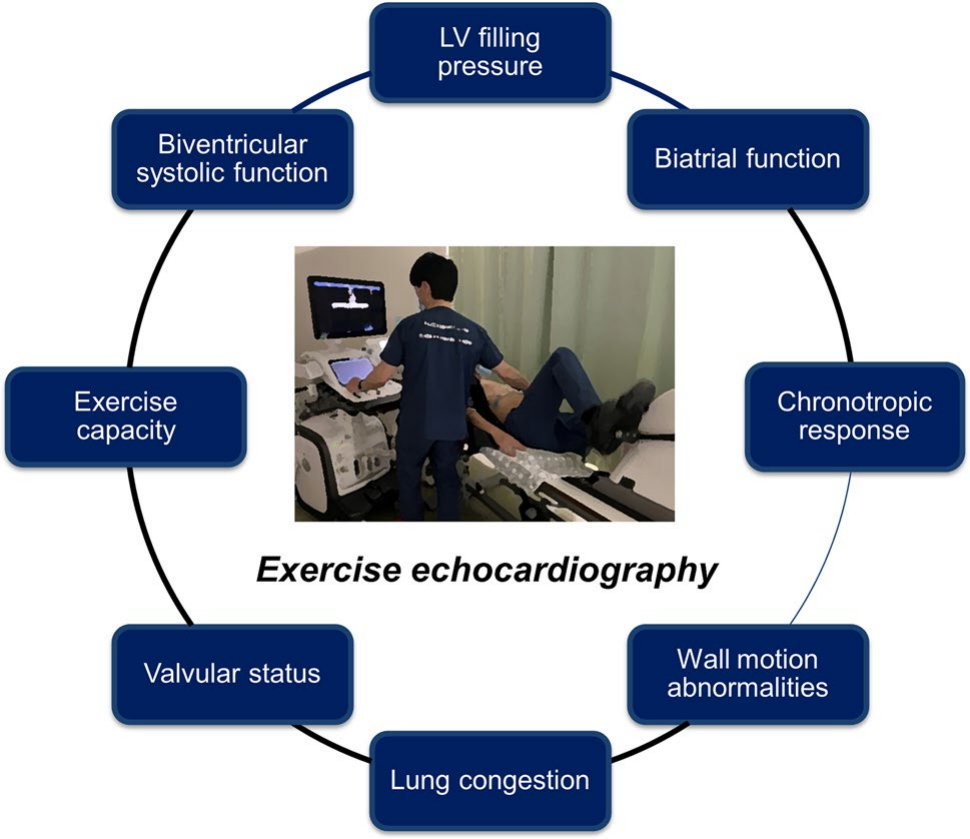
Potential utility of exercise stress echocardiography. Beyond the assessment of LV diastolic dysfunction and reserve, exercise stress echocardiography may provide valuable information regarding biventricular systolic function, wall motion abnormalities, biatrial function, pulmonary pressures, chronotropic response, cardiac output reserve, and valvular status during exertion

Beyond the LV diastolic dysfunction and reserve, exercise stress echocardiography provides valuable information regarding biventricular systolic function, wall motion abnormalities, biatrial function, pulmonary pressures, chronotropic response, cardiac output reserve, and valvular status during exertion (Fig. 3) [12, 13, 55, 58–61]. In particular, recent interest has focused on abnormal right ventricular-pulmonary artery interaction during exercise in patients with HFpEF [58, 62]. Previous studies have demonstrated that RV systolic and diastolic reserve function is impaired even in early-stage HFpEF, and this abnormality can be identified by exercise stress echocardiography [63]. The pathophysiological and prognostic significance of assessing RV contractile reserve limitation in the setting of worsening PH during exercise requires further investigation.

Performing cardiopulmonary exercise testing simultaneously with exercise echocardiography (CPETecho) allows detailed assessment of exercise capacity, ventilatory function, and peripheral oxygen uptake [58, 61, 64]. Although not yet standardized, it shows great promise in the assessment of HFpEF [65, 66]. In particular, sarcopenic and physical frailty are common in Japanese patients with HFpEF; therefore, measurement of peripheral oxygen uptake (arteriovenous oxygen content difference) may have pathophysiologic and therapeutic implications for HFpEF. In-depth characterization of patients using CPETecho may hold promise for the personalization of treatment for HFpEF (i.e., phenotyping) [67].

### Dyspnea clinic in the heart failure pandemic era

**Fig. 4:**
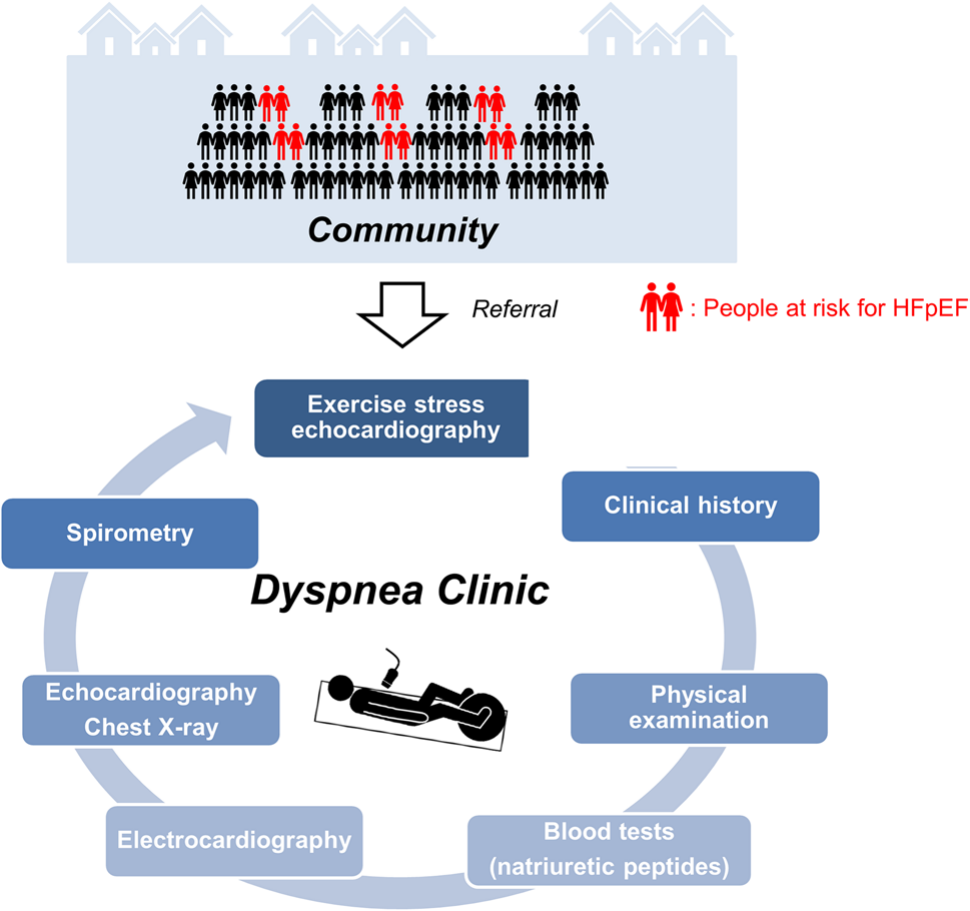
A dedicated dyspnea clinic to boost referrals from the community. A dedicated dyspnea clinic that consists of multi-step work-up including exercise stress echocardiography and cardiopulmonary exercise testing may boost referrals from primary care physicians in the community. This may allow early identification of HFpEF among patients with dyspnea at high risk of HFpEF. Abbreviations are the same as those in Fig. 1

As noted in the introduction, HF has become a pandemic, and a major contributing factor is the increasing prevalence of HFpEF [2, 3]. The increasing proportion of individuals with HFpEF and the emergence of effective disease-modifying therapies, such as SGLT2 inhibitors and glucagon-like peptide-1 receptor agonists, makes the accurate and timely identification of HFpEF even more important [68–70]. Dedicated dyspnea clinics, consisting of a multidisciplinary workup including CPETecho, are gaining attention as a way to identify early HFpEF and investigate the cause of unexplained dyspnea (Fig. 4) [64, 71]. As defined, the diagnosis of HFpEF primarily requires measurement of EF using echocardiography, which often requires referral by primary care physicians to secondary or tertiary hospitals. The presence of a dyspnea clinic may boost HFpEF referrals from the community. Furthermore, it may facilitate diagnosis and differentiation from other cardiac and non-cardiac diseases.

## Conclusion and future directions

We now understand the difficulty of diagnosing HFpEF in patients with less congestion and the potential clinical utility of exercise echocardiography to identify it. The next step will be to identify how to utilize exercise echocardiography in this era of the HF pandemic. One possible approach could be early diagnosis and treatment of HFpEF in dedicated dyspnea clinics. However, there are many unanswered questions and knowledge gaps with regard to exercise stress echocardiography in the evaluation and management of HFpEF (Table 1) [72–74]. Further studies are definitely warranted to improve quality of life and clinical outcomes for our patients.

**Table 1:** Key questions and knowledge gaps with regard to exercise stress echocardiography in HFpEF

Key questions	Gaps in evidence and future studies needed
Exercise stress echocardiography allows early identification of HFpEF among patients with dyspnea; however, it is unclear whether early diagnosis will improve the clinical outcomes	A retrospective observational study has shown that initiation of guideline-directed medical treatment after early diagnosis of HFpEF based on exercise stress echocardiography is associated with lower rates of all-cause mortality and worsening HF events (7); however, randomized control studies are needed to determine if intervention after an early diagnosis will improve clinical outcomes
No universally adopted protocols exist	It is unclear what exercise protocols cardiologists use in clinical practice. Further studies are needed to develop optimal protocols
Two diagnostic algorithms from professional societies are available (14, 26); however, these are based on expert consensus. Which of these criteria do cardiologists use in clinical practice to diagnose HFpEF? What is the optimal criterion for the diagnosis of HFpEF using exercise stress echocardiography?	There are no evidence-based criteria for the diagnosis of HFpEF using exercise echocardiography. Further investigation is needed to find out what problems cardiologists have in diagnosing HFpEF in clinical practice. Further studies are warranted to develop evidence-based diagnostic criteria
What is the role of exercise echocardiography with simultaneous cardiopulmonary exercise testing (CPETecho) in HFpEF? Is CPETecho useful for HFpEF phenotyping?	Although cardiopulmonary exercise testing provides an objective assessment of exercise capacity and risk stratification, its diagnostic value in identifying HFpEF in patients with symptoms of exertional dyspnea may be modest (61, 74). Further studies are needed to determine the clinical value of CPETecho in the management and better characterization of HFpEF
There are two schemes to assess the probability of HFpEF: the H_2_FPEF score and the HFA-PEFF algorithm (32). What is the optimal approach to identify patients at high risk of HFpEF?	There are considerable differences in clinical characteristics between Western and Japanese patients with HFpEF, such as the prevalence of obesity (33). Further studies are required to validate these scores in Japanese patients. Ideally, a scoring system to calculate the probability of HFpEF should be developed for Japanese patients
Previous studies have demonstrated the potential utility of artificial intelligence for HFpEF phenotyping (72, 73). How can it be used in the field of exercise stress echocardiography?	Applying artificial intelligence technology to exercise stress echocardiography results may hold promise for improving the accuracy of diagnosis and better characterizing patients' pathophysiology. Further studies are needed
HFpEF is a pathophysiologically heterogeneous syndrome. There may be differences in the efficacy of exercise stress echocardiography between different phenotypes or etiologies	Studies investigating the clinical value of exercise echocardiography in different etiologies of HFpEF are lacking. Further studies are needed to pursue this

*CPETecho* exercise stress echocardiography with simultaneous cardiopulmonary exercise testing, *HFA-PEFF* the heart failure association pre-test assessment, Echocardiography and natriuretic peptide, Functional testing, Final etiology, *HFpEF* heart failure with preserved ejection fraction
